# Gene expression profiles during tissue remodeling following bladder outlet obstruction

**DOI:** 10.1038/s41598-021-92756-1

**Published:** 2021-06-23

**Authors:** Saya Ito, Takeshi Nomura, Takashi Ueda, Shogo Inui, Yukako Morioka, Hisashi Honjo, Ayako Fukui, Atsuko Fujihara, Fumiya Hongo, Osamu Ukimura

**Affiliations:** Department of Urology, Graduate School of Medical Science, Kyoto Prefectural University of Medicine, Kyoto-City, Kyoto, 602-8566 Japan

**Keywords:** Bladder, Mechanisms of disease

## Abstract

Bladder outlet obstruction (BOO) often results in lower urinary tract symptoms (LUTSs) and negatively affects quality of life. Here, we evaluated gene expression patterns in the urinary bladder during tissue remodeling due to BOO. We divided BOO model rats into two groups according to the degree of hypertrophy of smooth muscle in the bladder. The strong muscular hypertrophy group, which exhibited markedly increased bladder smooth muscle proportion and *HIF1α* mRNA levels compared with the control group, was considered a model for the termination of hypertrophy, whereas the mild muscular hypertrophy group was considered a model of the initiation of hypertrophy. Some genes related to urinary function showed different expression patterns between the two groups. Furthermore, we found that several genes, including D-box binding PAR bZIP transcription factor (*DBP*), were upregulated only in the mild muscular hypertrophy group. *DBP* expression levels were increased in bladder smooth muscle cells in response to hypoxic stress. DBP associated with enhancer and promoter regions of *NOS3* gene locus and upregulated *NOS3* gene expression under hypoxic conditions. These findings suggested that the regulatory systems of gene expression were altered during tissue remodeling following BOO. Furthermore, circadian clock components might be involved in control of urinary function via transcriptional gene regulation in response to hypoxic stimuli.

## Introduction

Bladder outlet obstruction (BOO) occurs when there is a blockage at the base or neck of the bladder that inhibits the flow of urine into the urethra^1^. BOO is often observed in cases of benign prostatic hyperplasia (BPH) and can result in lower urinary tract symptoms (LUTSs), i.e., storage disturbances (e.g., daytime urinary urgency and nocturia) and voiding disturbances (e.g., urinary hesitancy, weak stream, straining, and prolonged voiding)^1^. LUTSs affect an estimated 30% of men over 85 years old in the United Kingdom and decreases quality of life^2^.

In addition to LUTSs, BOO also leads to progressive tissue remodeling of the bladder through three sequential stages: hypertrophy, compensation, and decompensation^3^. In the hypertrophy stage, mechanical stress causes hypertrophy of smooth muscle cells (SMCs) and hypoxia, resulting in stimulation of angiogenesis. In the compensation stage, bladder growth and angiogenesis are arrested. In the decompensation stage, loss of smooth muscle, deposition of extracellular matrix, and degradation of neurons occur under the sustained stress caused by obstruction. In each stage of the bladder remodeling, several signaling pathways are modulated in the various compartments of the bladder in animal models of BOO and human patients with BOO^3,4^. Alterations in gene expression at the initiation of bladder remodeling play important roles as triggers of disease progression. Hypoxia is one type of stress stimulus that induces upregulation of hypoxia-inducible factor (HIF) 1α and vascular endothelial growth factor (VEGF) in SMCs of the bladder^5^. An in vitro study using normal human bladder smooth muscle cells demonstrated that transcripts of *HIF1a* and *VEGF* are transiently increased in response to hypoxia in a time-dependent manner (i.e., HIF1α is transiently upregulated after 2 h, whereas *VEGF* is gradually upregulated after 24 h)^6^. However, the mechanisms regulating gene expression at the initiation of bladder remodeling in BOO have not yet been clarified.

In mammals, various urinary functions exhibit circadian variations. Circadian rhythms are controlled by several transcriptional factors encoded by clock genes; these factors sequentially regulate the expression of target genes^7^. Previous studies using mice with knockout or mutant clock genes have suggested that circadian clock components are involved in controlling urinary functions^8,9^. However, the mechanisms through which clock components mediated urinary functions have not been fully elucidated. Furthermore, the effects of BOO on the roles of clock components in urinary functions are unclear.

In this study, we aimed to elucidate the mechanisms regulating gene expression at the initiation of bladder tissue remodeling in BOO. Our findings demonstrated that gene expression patterns were altered during BOO-dependent bladder remodeling. Furthermore, we found that D-box binding PAR bZIP transcription factor (DBP), a circadian clock component, was upregulated in response to hypoxic stress and functioned as a regulator of a urinary function-related gene.

## Materials and methods

### Experimental animals

Eleven-week-old female Sprague–Dawley rats (230–250 g, n = 9) were housed with free access to food and water in a room with a 12-h light/dark cycle. Three control rats underwent a sham operation, and 6 rats underwent urethral constriction, as described previously^10,11^, with some modifications. To contract the bladder tissue, urine was drained from rat’s bladder by pressing the abdomen after induction of anesthesia with a mixture of medetomidine, midazolam, and butorphanol. Then, a midline abdominal incision was made. The proximal urethra was dissected with the bladder and surrounding tissue and was tied using silk ligature. A PE-90 catheter was placed beside the proximal urethra and was removed after the urethra was tied. In sham operation, we dissected the proximal urethra of anesthetized rats and closed the abdomen without any treatment. Animals were housed for an additional 4 weeks and then used for histological and gene expression analyses. All animal procedures were approved by the Animal Care and Use Committee of Kyoto Prefectural University of Medicine before the experiment and performed in accordance with the Guidelines for Animal Care of Kyoto Prefectural University of Medicine. The study was carried out in compliance with the ARRIVE guidelines.

### Hematoxylin and eosin (HE) stain and Immunohistochemistry

Bladder tissue was dissected from 3 bladders in the control group and 6 bladders in the BOO groups. Frozen sections were prepared by standard methods. Tissues were stained with hematoxylin and eosin. Immunohistochemistry was performed using Dako LSAB + system-HRP (Agilent, CA, USA) according to the manufacturer’s instructions. Rabbit anti-DBP antibodies (cat. No. 12662–1-AP; Proteintech, IL, USA; 1:50) were used. All images are obtained using AdvanView imaging software included in AdvanCam-E3Rs/ALL-IN-ONE system (AdvanVision, Tokyo, Japan). To indicate area of smooth muscle cells, number of cell nuclei in a single muscle bundle was measured using ImageJ. At the same time, the area of each muscle bundle was quantitated using ImageJ. [The muscle bundle area/number of cross-sectional nuclei] was calculated and determined as the SMC area. To indicate the rate of DBP-positive cells in immunostaining images, each of DBP-positive cells and total cells per area were quantified using ImageJ. [number of DBP-positive cells /number of total cells] was calculated and shown as rate of DBP-positive cells.

### RNA-seq

Preparation of sequencing libraries and Illumina sequencing were carried out at Novogene Bioinformatics Institute (Beijing, China). Total RNA from 3 bladders in the control group and 6 bladders in the BOO groups was isolated using ISOGEN (Nippon Gene, Tokyo, Japan) according to the manufacturer's instructions. RNA concentration was measured using Thermo Scientific NanoDrop (ThermoFisher, MA, USA). Then, RNA degradation and contamination were monitored by agarose gel electrophoresis. Moreover, the RNA integrity was assessed using the Agilent Bioanalyzer 2100 system.

Sequencing libraries were built using the NEBNext Ultra RNA Library Prep Kit for Illumina (NEB, MA, USA) according to the manufacturer’s instructions. Briefly, mRNA was purified from the total RNA using oligo dT attached magnetic beads and fragmented randomly by adding fragmentation buffer. First strand cDNA was synthesized using the mRNA template, a random hexamer primer and reverse transcriptase. Second strand cDNA synthesis was subsequently performed using dNTPs, RNase H and DNA polymerase I. After a series of terminal repair of the double-stranded cDNA, NEBNext Adaptor with a hairpin loop structure was ligated to prepare for hybridization. After purification with the AMPure XP beads (Beckman Coulter, Beverly, USA), library fragments were size-selected using USER Enzyme and then enriched by PCR using HiFi DNA polymerase and Index/Universal PCR primers. Finally, the library quality was assessed on the Agilent Bioanalyzer 2100 system.

The library preparations were sequenced on an Illumina NovaSeq 6000 platform, and 150 bp paired-end reads were generated. Clean data were obtained by removing reads containing adapter sequences, reads with more than 10% N, and reads containing low quality base from raw data recorded in a FASTQ file. Furthermore, quality of clean data such as Q20, Q30, GC-content, and sequence duplication level was calculated. Transcripts per million (TPM) counts obtained from high quality clean data were used as transcript expression levels. Gene Ontology (GO) analysis was performed using the PANTHER Gene List Analysis tool (http://www.pantherdb.org/).

### Cell culture and transfection

Primary bladder smooth muscle cells (BdSMC) were provided by Lonza and cultured using Reagent Pack (Lonza, Basel, Switzerland) at 37 °C under 5% CO_2_. To induce hypoxia in cell culture, CoCl_2_ (Nacalai Tesque) was used at a final concentration of 500 mM in culture medium. Cells were incubated in CoCl_2_-containing medium for 4–24 h at 37 °C in an atmosphere containing 5% CO_2_.

To knockdown DBP expression, transfection of siRNA targeting DBP (cat. no. SI00359751; Qiagen) and siRNA negative control (cat. no. 452002; Invitrogen, CA. USA) was performed using Lipofectamine RNAiMAX (Invitrogen) for 24 h.

### Reverse transcription quantitative polymerase chain reaction (RT-qPCR)

The following experiments were performed using kits according to the manufacturer's instructions. Total RNA was isolated using ISOGEN (Nippon gene, Toyama, Japan). RT was performed using PrimeScript RT Master Mix (Takara, Shiga, Japan). cDNAs were quantified by real-time PCR using SYBR qPCR mix (Toyobo, Osaka, Japan) and a Thermal Cycler TP800 (Takara). Primer sets for qPCR are shown in Table S2.

### Western blotting

Whole-cell lysates were subjected to sodium dodecyl sulfate (SDS)–polyacrylamide gel electrophoresis and then western blotting using standard methods^12^. Primary antibodies targeting DBP (cat. no. 12662-1-AP; Proteintech; 1:500), HIF1α (cat. no. H1a67, 400,080; Calbiochem, MA, USA; 1:500), and β-actin (cat. no. A5441; Sigma, MO, USA; 1:1000) were used.

### Chromatin immunoprecipitation (ChIP) assay

ChIP assays were performed using previously reported protocols^13^. Cells were fixed with 1% formalin for 10 min at 37 °C. Cell pellets were diluted with 10% SDS, 10 mM EDTA (pH 7.9), and 50 mM Tris–HCl (pH 8.1) and sonicated with an ultrasonic generator (UR-21P, TOMY, Tokyo, Japan) at 4 °C to shear chromatin to an average length of about 1 kb. The cell lysates were treated with IgG antibody (cat. no. I5006; Sigma) or DBP antibody (cat. no. 12662-1-AP; Proteintech) at 4 °C for 18 h after which Protein G magnetic beads (Bio-Rad) were added at 4 °C for 30 min. The beads were washed sequentially with (1) low salt buffer (0.1% SDS, 1% Triton X-100, 2 mM Tris–HCl, 250 mM NaCl), (2) high salt buffer (0.1% SDS, 1% Triton X-100, 2 mM Tris–HCl, 500 mM NaCl), (3) LiCl buffer (0.25 M LiCl, 1% NP40, 1% deoxycholate, 1 mM EDTA, 10 mM Tris–HCl) and finally (4) TE buffer (10 mM Tris–HCl, 1 mM EDTA). The washed beads were incubated with 10 mM Tris–HCl, 0.3 M NaCl, 5 mM EDTA, 0.5% SDS for 4 h at 65 °C. After incubation with RNaseA and Proteinase K, immunoprecipitated DNA fragments were amplified with several primer sets and quantified with Thermal Cycler TP800 (Takara). ChIP primers are shown in Table S3.

### Statistical analysis

Statistical analyses utilized *t*-tests or ANOVA as appropriate. A *P* value < 0.05 was considered significant. Information of statistical measures was mentioned in legends of each figure.

## Results

### Expression levels of genes involved in bladder function depended on the degree of tissue remodeling

**Figure 1:**
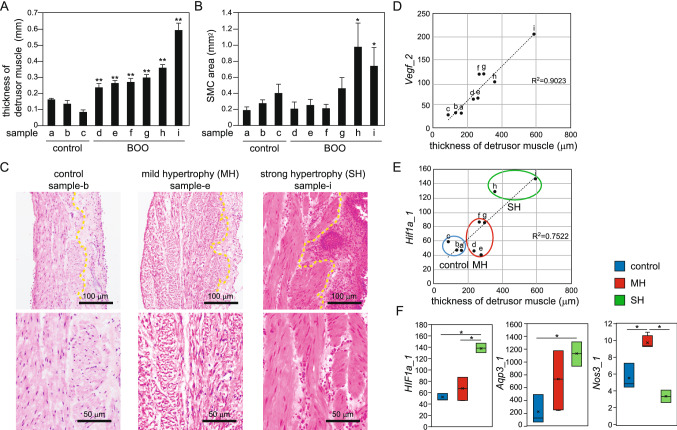
Expression patterns of genes related to hypoxia and urinary function in three experimental groups. (**A**) The thickness of the detrusor muscle in rats in the BOO model. Tissue thickness is shown as the average of 6 spots with a distance of 0.1 mm in the extra layer of muscle. Control rats underwent sham operation. Data are means ± SEM. A significant deferent test for sample-**a** is shown. **P < 0.01. (**B**) Cell area of smooth muscle in the BOO model rats. Number of cell nuclei in a single muscle bundle and the area of muscle bundle were measured using ImageJ. [The muscle bundle area/number of cross-sectional nuclei] was calculated and shown as the average of 4 bundles. Data are means ± SEM. A significant deferent test for sample-**c** is shown. *P < 0.05. (**C**) HE staining of the urinary bladder tissue in rats in the BOO model. HE stained images are data of samples shown in Fig. 1A and 1B. Yellow dotted lines in upper panels show borders between smooth muscle and lamina propria. Bottom panels show magnification of the detrusor muscle from each upper panel. All images are obtained using AdvanView 3.7 imaging software included in AdvanCam-E3Rs/ALL-IN-ONE system (http://advan-vision.com). (**D**) Correlations between *Vegf* levels and the thickness of the detrusor muscle in each rat (n = 9). *Vegf* mRNA levels were quantified using RNA-seq (TPM counts are shown on the vertical axis). Tissue thickness is the average value of 6 spots in the extra layer of muscle (horizontal axis). The letters above each dot corresponds to sample name in Fig. (**A**) and (**B**). (**E**) Correlations between *Hif1α* levels and the thickness of the detrusor muscle in each rat. (**F**) Expression patterns of genes related to urinary function in the control, MH, and SH groups. For each box plot, the box represents the 25th to 75th percentile interval, and the line represents the median. The cross mark represents average. Top or bottom of bar extending from the box represents the maximum or minimum, respectively. *P < 0.05.

To investigate gene expression profiles of bladder remodeling due to BOO, we generated an experimental BOO model in rats. The proportion of bladder smooth muscle increased markedly in all rats exposed to experimental partial outlet obstruction compared with those in control rats (Fig. 1A,C). Variations in the degree of muscular thickening were observed among rats, and rats with marked degree of muscular thickening showed statistically significant increase of cell area of bladder smooth muscle (sample h and i in Fig. 1B). We categorized BOO model rats into two groups according to the degree of hypertrophy of smooth muscular cells : the strong muscular hypertrophy (SH) group (sample h and i) and the mild hypertrophy (MH) group (sample d, e, f and g). The reason for the small population especially in the SH group was that many rats died due to experimental partial outlet obstruction [mortality after 4 weeks of operation of urethral constriction was 38.2% (n = 34)]. We then analyzed total gene expression patterns in the groups by RNA-seq (Table S1). We first confirmed the mRNA expression levels of the hypoxia markers *Vegf* and *Hif1α*. *Vegf* levels were significantly higher in all BOO rats than in control rats and were positively correlated with the thickness of the detrusor muscle (r^2^ = 0.9023, Fig. 1D). In contrast, *Hif1α* levels significantly increased only in SH group rats (Fig. 1E,F). HIF1α protein is known to be transiently upregulated in hypoxia, whereas *HIF1α* mRNA is frequently downregulated^14–16^. The reduction of *HIF1α* mRNA expression in hypoxia is an important component of the cellular adaptation to hypoxia. Therefore, SH group rats exhibiting high levels of *Hif1α* mRNA were presumed to be in the termination of hypertrophy stage and to have lost protection against hypoxia. In contrast, MH group rats exhibiting low levels of *Hif1α* mRNA were thought to be in the initiation of hypertrophy stage.

Next, we compared the expression patterns of some genes involved in bladder functions from RNA sequencing results between the three groups. Aquaporin 3 (AQP3) is an intrinsic membrane protein belonging to the aquaporin family and functions as a water channel in many cell types involved in fluid transport^17^. Nitric oxide synthase 3 (NOS3) is an endothelial nitric oxide synthase that is involved in the induction of apoptosis in the bladder mucosa^18^. *Aqp3* was markedly upregulated in the SH group compared with those in the control group and the MH group (Fig. 1F). In contrast, *Nos3* was increased only in the MH group (Fig. 1F). These results suggested that the expression levels of some genes related to bladder function were altered during bladder remodeling following BOO. Furthermore, the expression patterns were different for every gene.

### Total gene expression profiles in the MH and SH groups

**Figure 2:**
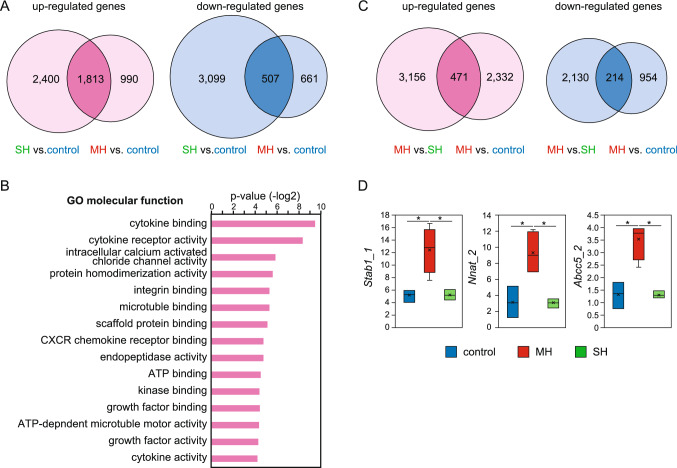
Total gene expression patterns in the three experimental groups. (**A**) Gene expression profiles of urinary bladder tissues from the control, MH, and SH groups using RNA-seq results. Comparison analysis was performed between the SH versus control and MH versus control groups. In total, 1,813 genes exhibited more than twofold increases in mRNA expression in the MH and SH groups compared with the control group, whereas 507 genes were downregulated by at least 50% in the MH and SH groups. (**B**) RNA-seq-based GO analysis of genes upregulated more than twofold in the MH and SH groups compared with the control group (1,813 genes shown as dark pink in Fig. **A**). (**C**) Gene expression profiles with comparisons between the MH and SH groups and the MH and control groups. In total, 471 genes exhibited a more than twofold increase in mRNA expression in the MH group compared with the SH and control groups. Additionally, 214 genes showed a more than twofold decrease in expression in the MH group compared with the other two groups. (**D**) Expression pattern of each gene showing marked changes in mRNA expression in the MH group compared with the SH and control groups. The box represents the 25th to 75th percentile interval, and the line represents the median. The cross mark represents average. Top or bottom of bar extending from the box represents the maximum or minimum, respectively. *P < 0.05.

To compare total gene expression patterns between the MH and SH groups, we further analyzed the results of RNA sequencing. In total, 4,213 genes exhibited a more than twofold increase in expression in the SH group compared with those in the control group, whereas 2,803 genes were increased in the MH group (Fig. 2A). Among these upregulated genes, 1,813 genes were upregulated in both the SH and MH groups (Fig. 2A). In contrast, 507 genes exhibited less than half the expression in both the SH and MH groups compared with the control group (Fig. 2A). The 1,813 upregulated genes in both the MH and SH groups were categorized using GO analysis (Fig. 2B). The results showed that many of the genes were categorized into the molecular functions category, including cytokines and cytokine receptors (Fig. 2B), consistent with the results of previous studies using BOO model rats^4,19^.

Next, we focused on genes showing alterations in expression levels only in the MH group. We showed that 471 genes exhibited a more than twofold increase in expression in the MH group compared with those in the control and SH groups, whereas 214 genes in the MH group were expressed at levels less than half those in the other two groups (Fig. 2C). Some genes, including stabilin 1 (*Stab1*), ATP binding cassette subfamily C member 5 (*Abcc5*), and neuronatin (*Nnat*) were markedly increased in the MH group compared with those in the control and SH groups (Fig. 2D). Genes showing altered mRNA expression levels may play roles in bladder remodeling at the initiation of the hypertrophy stage.

### *Dbp* was highly expressed in the MH group

**Figure 3:**
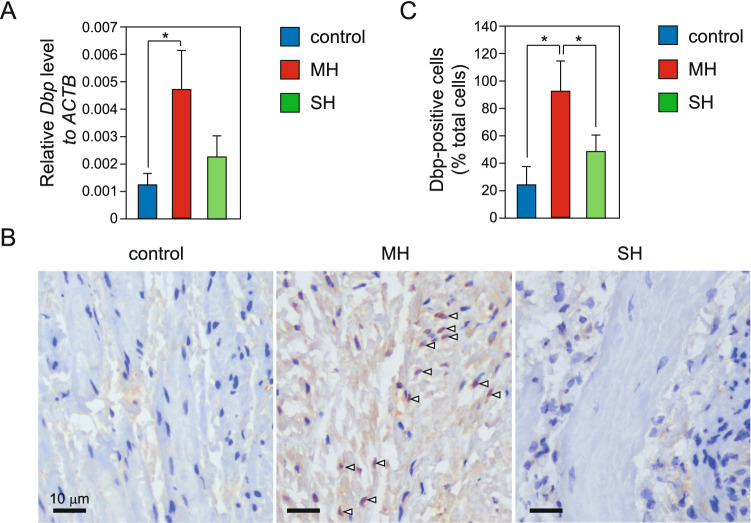
Expression levels of *Dbp* in the three experimental groups. (**A**) Relative *Dbp* mRNA levels in the rat urinary bladder in the control, MH, and SH groups. mRNA levels were quantified by RT-pPCR. Measurements were normalized to *Actb* mRNA levels. Data are means ± SEM. *P < 0.05. (**B**) Immunohistochemistry using anti-DBP antibodies. DBP was stained brown via DAB immunolabeling (white arrowheads). Nuclei were stained blue by hematoxylin stain. All images are obtained using AdvanView 3.7 imaging software (http://advan-vision.com). Scale bars: 10 mm. (**C**) The number of DBP-positive cells in immunostaining images was quantified using ImageJ. Data are means ± SEM (n = 4). *P < 0.05.

DBP was identified as one of the genes whose expression was increased in the MH group, although there was no statistically significant difference in RNA-seq results (Fig. S1). DBP is a transcriptional factor belonging to the circadian clock family and plays roles in the control of circadian rhythm and clock outputs via regulation of target gene expression^20^. Thus, we hypothesized that DBP may regulate the expression of genes involved in bladder functions at the initiation of bladder remodeling due to BOO. First, to confirm the expression levels of *Dbp* in rat BOO models, we quantified *Dbp* mRNA levels by RT-qPCR. *Dbp* mRNA levels were significantly increased in the MH group than in the control group (Fig. 3A). We then assessed DBP protein expression by immunohistochemistry using anti-DBP antibodies. DBP protein was detected in the nuclei of most bladder smooth muscle cells in the MH group, but scarcely in the control and SH groups (Fig. 3B,C).

### DBP upregulated *NOS3* gene expression under hypoxic conditions in human urinary bladder cells

**Figure 4:**
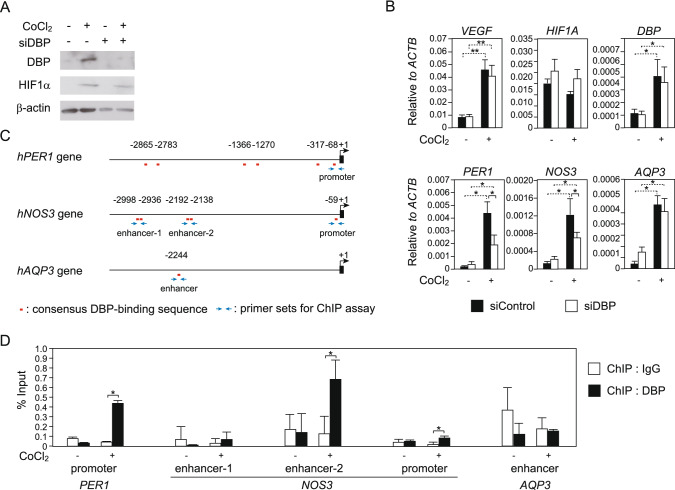
DBP-mediated gene expression under hypoxic conditions in human bladder smooth muscle cells. (**A**) DBP protein levels in human bladder smooth muscle cells under hypoxic conditions. BdSMC cells were transfected with siDBP and cultured with CoCl_2_ solution for 24 h (0 or 500 mM). Western blotting was performed with anti-DBP and anti-HIF1α antibodies. (**B**) mRNA levels of DBP-knockdown BdSMC cells under hypoxic conditions. BdSMC cells were transfected with siDBP and treated with CoCl_2_ for 24 h. RT-qPCR was used to measure gene expression. Measurements show average values of 3 independent measurements, which were normalized to *ACTB* mRNA expression. **P < 0.01, *P < 0.05. (**C**) A schematic of gene loci of human *PER1*, *NOS3* and *AQP3*. Locations of DBP-binding sequences are shown as red bars. Blue arrows indicate primers for ChIP assay. (D) ChIP assay with anti-DBP antibody using BdSMC cell lysate. BdSMC cells were cultured with 500 mM CoCl_2_ solution for 0 h or 4 h. Immunoprecipitated DNA fragments were quantified by qPCR. Each primer set is shown in Table S3. Data are means ± SEM for three independent experiments. *P < 0.05.

Next, we assessed whether DBP was upregulated under hypoxic conditions in human bladder cells. Hypoxia was induced in human primary bladder smooth muscle cells by treatment with cobalt chloride (CoCl_2_) solution. Protein levels of the hypoxia marker HIF1α were increased by CoCl_2_ (Fig. 4A). *VEGF* mRNA levels were markedly increased by CoCl_2_, whereas *HIF1α* mRNA levels were not changed (Fig. 4B). This condition caused by CoCl_2_ was considered to reflect the initial hypoxia. Under the hypoxic condition, protein and mRNA levels of DBP were higher than those under normal conditions (Fig. 4A,B).

To investigate whether DBP contributed to bladder functions under hypoxic conditions, we examined knockdown of DBP in bladder smooth muscle cells using siRNA for DBP. DBP protein levels were efficiently decreased by siDBP in bladder smooth muscle cells (Fig. 4A), although DBP mRNA levels were scarcely changed (Fig. 4B). A previous report suggested that DBP protein positively regulates period circadian regulator 1 (*PER1*) promoter, while PER1 protein negatively regulates *DBP* gene expression^20^. Based on the above findings, decrease of DBP mRNA caused by DBP knockdown might be countered by dysregulation of PER1-mediated negative feedback loop. mRNA levels of the *PER1* gene were increased by CoCl_2_ and decreased by knockdown of DBP under hypoxic condition (Fig. 4B). These results suggested that DBP was increased in response to hypoxia and controlled gene expression of its target genes via transcriptional regulation in human bladder cells. Next, we quantified mRNA levels of genes related to bladder function (*NOS3* and *AQP3*) using DBP-knockdown cells. The RNA-seq analysis using BOO model rats had shown that rat homolog of *NOS3* and *AQP3* genes were markedly upregulated in the MH group and the SH group, respectively (Fig. 1F). In human bladder cells, mRNA levels of *NOS3* and *AQP3* were increased by treatment with CoCl_2_ (Fig. 4B). The increase in *NOS3* under hypoxic conditions were blocked by *DBP* knockdown; however, the expression levels of *AQP3* were not affected (Fig. 4B).

To assess whether DBP controlled expression of *NOS3* gene via transcriptional regulation, we investigated binding of DBP to the gene locus of *NOS3* by ChIP assay using anti-DBP antibodies. Consensus DBP-binding sequences (5’-RTTAYGTAAY-3’) are located in *PER1*, *NOS3* and *AQP3* gene loci (Fig. 4C). DBP associated with an enhancer region located 2.1 kb upstream of the transcription start site (enhancer-2 in Fig. 4C) and the promoter region of *NOS3* gene under hypoxic condition in common with *PER1* gene promoter (Fig. 4D). On the other hand, DBP did not associate with a DBP-binding site located in *AQP3* gene locus with or without CoCl_2_ (Fig. 4D). Above results suggested that *NOS3* gene was transcriptionally activated by DBP under hypoxic conditions in bladder smooth muscle cells.

## Discussion

Urinary functions are controlled by several signaling pathways. In this study, our findings suggested that BOO modulated signaling pathways by regulation of gene expression in the bladder. Gene expression patterns were altered during tissue remodeling due to BOO. Furthermore, we found that DBP involved in urinary functions in response to hypoxic stress via transcriptional regulation of *NOS3* gene.

HIF1α is a key regulator of cellular response to hypoxia^21^. The expression levels of HIF1α are highly regulated, and HIF1α abundance is controlled through transcriptional, post-transcriptional, and post-translational mechanisms^21^. HIF1α protein is transiently upregulated in hypoxia but rapidly degraded through von Hippel-Lindau tumor suppressor protein-mediated protein degradation in normoxia^22^. In contrast, *HIF1α* mRNA is frequently suppressed, despite expression of HIF1α protein, under hypoxic conditions^14–16^. The reduction in *HIF1α* mRNA expression in hypoxia indicates that this molecule may be important for cellular adaptation to hypoxia, and this hypothesis is supported by the observation that high levels of *HIF1α* mRNA have been observed in some cancers and are often associated with poor prognosis^23–26^. Our experimental BOO model rats were subjected to intravesical obstruction for 4 weeks, and a part of individual rats showed increased *HIF1α* mRNA levels. Their smooth muscle contents were significantly increased, suggesting that these rats were in the termination of hypertrophy stage during tissue remodeling due to BOO. Thus, these results support that high levels of *HIF1α* mRNA indicate dysfunction of the cellular adaptation system to hypoxia in bladder tissue. mRNA levels of *HIF1α* can be a useful indicator of health status due to BOO and may facilitate prediction of prognosis in patients with cancer.

Although circadian rhythms are known to affect urinary function, the roles of circadian clock components in this process are not clear^8^. DBP participates in control of circadian rhythm and clock outputs via rhythmically activating the transcription of various genes through a DNA cis-element, the D-box^20^. In fact, previous studies with mice deleted for the *Dbp* gene have shown that DBP is involved in the regulation of several clock outputs, including locomotor activity, sleep distribution, and liver gene expression^27–29^. In this study, our findings demonstrated that DBP was upregulated in response to hypoxia in urinary bladders using BOO model rats and human primary cells. We further showed that DBP transcriptionally activated *NOS3* gene under hypoxic conditions. NOS3 is known as an endothelial nitric oxide synthase (eNOS) and synthesizes nitric oxide (NO) in endothelial cells^30^. NO plays a pivotal role in the physiology of urinary bladder (e.g., regulation of local arteriolar tone and smooth muscle relaxation)^31^. Furthermore, a previous study suggested that eNOS might be involved in eliminating hyperplastic urothelial cells by triggering apoptosis in response to obstructive stimuli^18^. Combining the past reports and our findings, DBP may be involved in defense mechanisms against bladder dysfunction due to BOO via control of eNOS levels. Additional studies such as identification of DBP target genes are important for improving our understanding of the roles of circadian clock components in the physiology of urinary bladder. Furthermore, the relationship of circadian rhythms with urinary function under hypoxic conditions and/or disease progression due to BOO may lead to the development of effective treatments for BOO.

To identify regulators of gene expression at the initiation of adaptation to hypoxia, we performed total gene expression analysis by RNA-seq using experimental BOO model rats. Some genes, including *Dbp*, were identified as candidate regulators exhibiting alterations in mRNA expression at the early stage of hypertrophy of SMCs following BOO. Further studies of these candidate regulators are needed to fully elucidate the molecular mechanisms involved in initiation of tissue remodeling due to BOO and to identify new therapeutic targets for management of LUTSs.

## Supplementary Information

Supplementary Information.
