# Supplementary material for: Gene expression profiles during tissue remodeling following bladder outlet obstruction
Source: Sci Rep. 2021 Jun 23;11:13171. doi: 10.1038/s41598-021-92756-1 (PMC8222387; doi:10.1038/s41598-021-92756-1)

Gene expression profiles during tissue remodeling following bladder outlet obstruction

Saya Ito[1,*], Takeshi Nomura[1], Takashi Ueda, Shogo Inui, Yukako Morioka, Hisashi Honjo, Ayako Fukui, Atsuko Fujihara, Fumiya Hongo, Osamu Ukimura

Department of Urology, Graduate School of Medical Science, Kyoto Prefectural University of Medicine, Kyoto-City, Kyoto 602-8566, Japan

[1]These authors equally contributed to this work.

[*]Corresponding author.

Saya Ito

Department of Urology, Graduate School of Medical Science, Kyoto Prefectural University of Medicine, Kyoto-City, Kyoto 602-8566, Japan

FAX: +81 75 251 5598

TEL: +81 75 251 5595

E-mail: itosaya@koto.kpu-m.ac.jp

Ito S et al., Figure S1

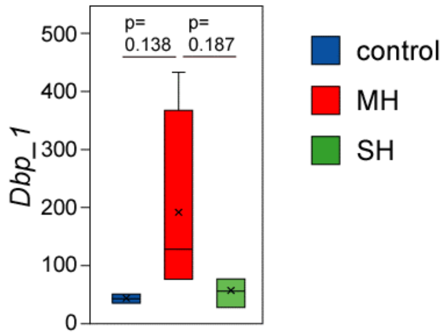

Figure S1. Expression levels of *Dbp* in the three experimental groups.

Expression patterns of *DBP* gene of urinary bladder tissues from the control, MH, and SH groups using RNA-seq results. For each box plot, the box represents the 25th to 75th percentile interval, and the line represents the median. The cross mark represents mean value. Top or bottom of bar extending from the box represents the maximum or minimum value, respectively.

	control			mild-BOO				strong-BOO	
Gene Name	a	b	c	d	e	f	g	h	i
Vegfa_2	26.86593566	31.29052131	31.46184725	62.67916646	66.061479	118.233951	118.233951	99.62412035	205.5101282
Hif1a_1	58.35806848	47.4891482	46.64246075	46.87245061	49.3991699	86.10624011	86.10624011	129.9365658	146.8060865
Aqp3_1	127.6326595	495.6807109	63.36214914	288.8617255	249.1139504	1177.506693	1177.506693	1320.157698	936.3127902
Nos3_1	4.573916751	4.935637546	7.333915079	11.04710944	9.574186109	9.393425638	9.393425638	2.568214604	4.027939174
Dbp_1	42.94989867	37.55364794	53.88611218	176.9975979	433.3771203	79.59366007	79.59366007	28.75594201	78.86824092
Stab1_1	3.974633404	5.032714174	5.641486749	7.238778441	16.31807387	12.72264351	12.72264351	5.852839361	4.216463679
Abcc5_2	1.426483695	1.815488322	0.773071942	2.395206497	3.621403818	3.948348744	3.948348744	1.187627718	1.473189059
Nnat_2	1.133201551	2.961717431	4.971527961	11.02390912	12.14255108	6.881264286	6.881264286	3.369478131	2.340608775

Table S1. Part of results of RNA-seq

Transcript expression levels from each rat are show as TPM counts.

gene		sequence
rDbp	forward	5'-AGACTTACACCTGACACCCC-3'
	reverse	5'-GATTGTGTTGATGGAGGCGG-3'
rActb	forward	5'-GTATGGAATCCTGTGGCATCC-3'
	reverse	5'-TAGAGCCACCAATCCACACA-3'
hHIF1A	forward	5'-AGCCGAGGAAGAACTATGAACATAA-3'
	reverse	5'-GTGGCCTGTGCAGTGCAA-3'
hVEGF	forward	5'-ATCCAATCGAGACCCTGGTG-3'
	reverse	5'-TGGTGATGTTGGACTCCTCA-3'
hDBP	forward	5'-TACAAGAACAACGAGGCAGC-3'
	reverse	5'-CTGGTATCGGGACAGCAC-3'
hPER1	forward	5'-TCTCAGAGCTGGATGGACTG-3'
	reverse	5'-TGCTGTAGGTAAGGCTGGAC-3'
hAQP3	forward	5'-CTTCACGATCCACCCTTTCAG-3'
	reverse	5'-GGCCTAAGGTGCTATTTGGG-3'
hNOS3	forward	5'-AGAACTCTTCCTTCTGCCCC-3'
	reverse	5'-AGGATGTTGTAGCGGTGAGG-3'
hACTB	forward	5'-GTGGCCGAGGACTTTGATTG-3'
	reverse	5'-GCAATGCTATCACCTCCCCT-3'

Table S2. Sequences of RT-qPCR primers

Oligonucleotide primers used for RT-qPCR analysis are shown.

gene		sequence
PER1 promoter	forward	5'-TGGAGACCACTAGCCAATCA-3'
	reverse	5'-GGATGGCCGCAGAGATGC-3'
NOS3 enhancer -1	forward	5'-TGGATGCCGCAGTCTAAGAT-3'
	reverse	5'-GGGAGGTGTTGAGATCGTGA-3'
NOS3 enhancer -2	forward	5'-AACCCTCAGCATCCCAGAAG-3'
	reverse	5'-CGGGGAAAGGAGGGAAGG-3'
NOS3 promoter	forward	5'-CCGGATTGGCAGGGTAAGTA-3'
	reverse	5'-CGTCTCCTTGCCCCTAGTTT-3'
AQP3 enhancer	forward	5'-CATGCCTGTAGTCCCAGCTA-3'
	reverse	5'-GATCAGCACCAAGAAAGCCC-3'

Table S3. Primer sets for ChIP assay

Sequences of oligonucleotide primers used for ChIP assay are shown.

Original blots:

Figure 4A

DBP HIF1A b-Actin

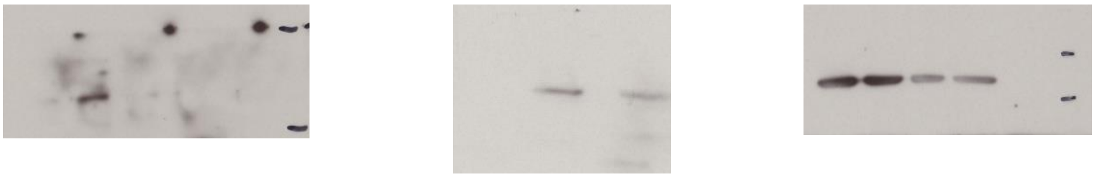

Supplement: Supplementary file 1 — Supplementary Information. [file 41598_2021_92756_MOESM1_ESM.pdf]
